# The influence of home environmental factors on kindergarten children’s addition strategy use

**DOI:** 10.3389/fpsyg.2022.1027431

**Published:** 2023-01-11

**Authors:** Mary DePascale, Susanne M. Jaeggi, Geetha B. Ramani

**Affiliations:** ^1^Department of Human Development and Quantitative Methodology, University of Maryland, College Park, College Park, United States; ^2^School of Education, University of California, Irvine, Irvine, United States

**Keywords:** addition strategy, SES, home learning environment, home literacy, home math environment, arithmetic, early childhood

## Abstract

Young children vary widely in their levels of math knowledge, their abilities to solve math problems, and the strategies they use to solve math problems. As much of later math builds on children’s early understanding of basic math facts and problem-solving strategies, understanding influences on children’s early problem solving is important. Few studies, however, have examined the home environment in relation to children’s strategy use during arithmetic problems. We examined how both structural characteristics of children’s home environments, such as socioeconomic status (SES), as well as the learning environment, such as engagement in math and literacy activities at home, related to their use of problem-solving strategies for numerical addition problems. Kindergarten children from diverse backgrounds completed a measure of addition problem solving and strategy use, including simple and complex numerical problems. Strategies were coded based on a combination of accuracy and strategy sophistication, with higher scores indicating problems solved correctly with more sophisticated strategies. Parents completed a home activities questionnaire, reporting the frequency with which they and their child had engaged in math and literacy activities at home over the past month. An exploratory factor analysis identified three components of the home activities - a basic activities factor, an advanced math activities factor, and a literacy activities factor. Findings indicated that SES related to children’s strategy sophistication, and frequency of engaging in advanced math and literacy activities at home predicted strategy sophistication, however, engaging in activities at home did not moderate the relations between SES and strategy sophistication. This suggests that family engagement in activities at home may promote early arithmetic skills, and that the role of home environmental characteristics should be considered in children’s arithmetic strategy use and performance over development.

## 1. Introduction

From a young age, children vary widely in their levels of math knowledge and their abilities to solve math problems. Early math knowledge is particularly important because it provides a foundation for and is predictive of later math development and academic achievement (Watts et al., 2014). Specifically, much of more complex math concepts build on children’s early understanding of basic math facts and problem-solving strategies. Therefore, understanding influences on children’s early problem solving is critical. Prior research suggests the importance of the home environment for children’s early math knowledge (Mutaf-Yıldız et al., 2020; Daucourt et al., 2021). However, few studies to date have examined the home environment in relation to children’s strategy use during arithmetic problems. The goal of the current study was to examine the role of home environmental factors in children’s addition strategy use.

The home environment includes both structural characteristics of the home, such as socioeconomic status (SES), as well as the home learning environment, such as engagement in math and literacy activities at home. Each of these aspects of the home environment can contribute to children’s early math development. For example, studies have shown that children from lower-income backgrounds may begin school at a lower level than children from higher-income backgrounds (Jordan et al., 2006, 2009). Reasons for this difference may include factors related to the home learning environment, such as access to resources and learning opportunities within the home, including engagement in learning activities at home (Laski et al., 2016; Daucourt et al., 2021).

In considering the home learning environment, studies have examined both the home literacy environment—a measure of families’ engagement in literacy activities, interactions, and beliefs at home—as well as the home math environment—a measure of families’ engagement in math activities, talk, and attitudes/beliefs at home. For both the home literacy and home math environments, it is theorized that parent attitudes about the subject area (i.e., literacy or math) and frequency of engaging in informal (e.g., games and playful activities) and formal (e.g., direct math or literacy activities, such as counting and reading) activities relate to children’s abilities in literacy and math (Skwarchuk et al., 2014). Recent meta-analyses and reviews have shown that the home math environment positively relates to children’s math development (Mutaf-Yıldız et al., 2020; Daucourt et al., 2021). For example, the frequency of parent and child engagement in early math activities such as counting on fingers, using number or quantity (e.g., more, less) words, and talking about simple math facts has been shown to relate to children’s math abilities in preschool and kindergarten (Blevins-Knabe and Musun-Miller, 1996; Anders et al., 2012; Vandermaas-Peeler and Pittard, 2014). In addition, children’s engagement in math games at home as preschoolers and kindergartners has been shown to relate to their concurrent math skills and predict their informal and formal math skills longitudinally through first grade (Niklas and Schneider, 2014; Zhang et al., 2020). Studies have also indicated that the home literacy environment positively relates to children’s math development, with the frequency of parent and child engagement in early literacy activities, such as reading books and identifying letters and letter sounds, relating to children’s early math and numeracy skills (Anders et al., 2012; Manolitsis et al., 2013). Engaging in literacy activities can support math skills through children’s development of vocabulary and language skills as well as through the home learning environment more broadly, as engagement in literacy activities may relate to engagement in numeracy activities (Anders et al., 2012; Manolitsis et al., 2013; Napoli and Purpura, 2018). Overall, these findings suggest that engaging in math and literacy activities at home can play an important role in early math development. Few studies, however, have examined the home environment in relation to children’s strategy use during arithmetic problems.

Arithmetic strategies are the types of problem-solving strategies children use when solving arithmetic problems. Strategies for simple addition problems include counting processes like using fingers or speaking out loud, as well as other mental methods for solving problems, such as automatic fact retrieval, guessing, or breaking down the problem into different parts (Geary et al., 2004). Strategies can be broken down into multiple levels of sophistication within finger and verbal counting, with more efficient strategies such as counting up from the largest addend in an addition problem (i.e., min strategy) viewed as more sophisticated than less efficient strategies such as counting up from the smaller addend (i.e., max strategy) or counting both addends (i.e., sum strategy). Even more sophisticated are strategies where children rely more on their memory and knowledge of addition facts. For example, children may use their knowledge of simple sums to break down a problem into smaller parts (i.e., recognizing that 2 + 5 is the same as 2 + 3 + 2). Children may also simply directly retrieve answers to specific problems from memory.

As children develop their arithmetic problem-solving skills, they vary in the strategies they use and tend to use multiple strategies to solve similar problems (Siegler, 1987, 1996). Throughout development, the strategies children use progress from being primarily simple strategies to more complex, memory and retrieval-based strategies (Ashcraft, 1982; Svenson and Sjöberg, 1983; Baroody, 1987; Geary et al., 1991; Paul and Reeve, 2016). This trajectory of development is critical for children’s development of increasingly complex math concepts and their problem-solving abilities, as the sophistication of children’s strategy choices relates to their later math performance, and becomes increasingly predictive of math performance longitudinally (Geary et al., 2017). In this way, having a strong foundation in early problem-solving abilities and being set on a trajectory of developing increasingly advanced problem-solving strategies is critical for later math development and achievement. However, previous research indicates that children’s development and use of strategies can vary based on personal and environmental factors, including children’s math abilities (Bailey et al., 2012), working memory abilities (Cragg and Gilmore, 2014), math anxiety (Ramirez et al., 2016), socioeconomic background (Laski et al., 2016), as well as whether problems are solved in an academic or play context (Bjorklund and Rosenblum, 2002; Bjorklund et al., 2004; Casey et al., 2020) and what materials are used for problem solving (Schiffman and Laski, 2018). Understanding the factors that influence this development is important for developing interventions to aid children in their math learning and development of problem-solving skills. The current study specifically focused on the role of home environmental factors to better understand the roles of SES, and the math and literacy activities children engage in at home on the development of children’s arithmetic strategies.

Research indicates that children from different socioeconomic backgrounds vary in their ability to solve simple and complex arithmetic problems. For example, Ginsburg and Pappas (2004) found that 4- and 5-year-old children from higher SES backgrounds performed better on addition problems than same-age peers from middle or lower SES backgrounds. Children from higher SES backgrounds were also more likely to use more sophisticated strategies, such as recall strategies, and less likely to use strategies such as touching and counting manipulatives to solve the problems. Similarly, Laski et al. (2016) found that kindergarten and first-grade students from higher-income backgrounds tended to use more sophisticated, efficient strategies, including decomposition, retrieval, and counting on from the larger addend. In contrast, students from lower-income backgrounds tended to use more inefficient strategies, including counting each addend before counting the total of both addends and other strategies. In addition, children from lower-income backgrounds were more likely to use simpler strategies as first graders than children from higher-income backgrounds. Results also indicated that children from higher-income backgrounds were more likely to solve problems accurately, and this relation of income with addition accuracy was mediated by use of sophisticated addition strategies.

These studies indicate that children’s SES background can influence their problem-solving strategies from a young age. It is possible that socioeconomic differences in the home environment, resources, and opportunities may contribute to these differences (Ginsburg and Pappas, 2004; Laski et al., 2016). Further understanding these influences on strategy use is important, because early strategy use is important for children’s later development of problem-solving and math abilities. The current study examines both overall strategy sophistication and frequency of use of individual strategies in relation to children’s SES backgrounds, as well as the role of the home learning environment in the relations between SES and arithmetic strategies.

Multiple studies have shown positive relations of children’s home numeracy experiences and their accuracy on addition problems. For example, parental reports of children’s engagement in home numeracy activities relate to their children’s single-digit addition problem fluency (LeFevre et al., 2009), and performance on symbolic (Dearing et al., 2012) and non-symbolic addition and subtraction (Skwarchuk et al., 2014). Another study indicated that children’s accuracy on single-digit non-symbolic arithmetic related to their engagement in math games at home, but did not relate to engagement in other home numeracy activities (Mutaf Yıldız et al., 2018).

Studies of children’s home literacy experiences have also shown positive relations of children’s home literacy experiences and their math abilities (Anders et al., 2012; Manolitsis et al., 2013; Napoli and Purpura, 2018). These studies suggest that engaging in activities that support language skills can support math development and that relations between engaging in home literacy and home math activities may also explain relations between literacy activities and math development (Anders et al., 2012; Manolitsis et al., 2013; Napoli and Purpura, 2018). However, results are also mixed, such that some studies do not show significant relations between the home literacy environment and children’s math abilities (LeFevre et al., 2009; Segers et al., 2015). Further, many studies examining relations between the home literacy environment and math abilities focus on math and numeracy skills more broadly (e.g., using broader measures that include multiple areas of early math skills), rather than examining relations with individual skills, such as arithmetic strategy use, directly.

Overall, these studies highlight the importance of the home learning environment and indicate that children’s math development is influenced by factors in their home environments. As these home factors are known to relate to children’s math skills in general, it is plausible that these same factors influence children’s developing understanding and use of addition strategies. The current study examines this by considering how children’s engagement in activities at home influences their addition strategy use.

The goal of the current study was to examine the role of the home environment in children’s addition strategy use. Specifically, we examined how both structural characteristics of children’s home environment, such as socioeconomic status (SES), as well as the learning environment, such as engagement in math and literacy activities at home, relate to their use of problem-solving strategies for numerical addition problems. The study contributes to the literature by examining the relation of children’s home activities to both accuracy and strategy use. Because the sophistication of children’s strategy use relates to their later math performance, and becomes increasingly predictive of math performance longitudinally (Geary et al., 2017), understanding factors that may influence children’s development and use of addition strategies is critical.

The first aim was to examine structural characteristics of children’s home environment in relation to their strategy use during arithmetic problem solving. We examined how SES related to children’s use of strategies to solve addition problems. We expected to replicate previous findings that income relates to strategy use, with children from higher-income backgrounds tending to use more efficient, sophisticated strategies, and children from lower-income backgrounds tending to use more inefficient strategies (Laski et al., 2016).

The second aim was to examine children’s home learning environment in relation to their addition strategy use. We examined how the frequency of children’s engagement in learning activities at home related to their use of strategies when solving addition problems. Engaging in more math activities, and specifically more activities related to mathematical problem solving, could provide children with more practice with basic math facts and enhance children’s problem solving, and therefore promote their use of more sophisticated addition strategies. We also examined relations between children’s addition strategy use and engagement in literacy activities at home, as these activities have the potential to support children’s mathematical skills as well (Anders et al., 2012; Manolitsis et al., 2013).

The third aim was to examine if home activities moderated the relations between socioeconomic status and children’s addition strategy use. Based on previous research examining relations of SES with children’s arithmetic skills (Laski et al., 2016), math skills, and home environment (Dearing et al., 2012; Galindo and Sonnenschein, 2015; Daucourt et al., 2021), we expected that the relations between SES and addition strategy use would vary based on the frequency of engaging in activities at home. Examining if home activities are a moderator of these relations could provide information for future interventions for promoting children’s arithmetic skills.

## 2. Materials and methods

### 2.1. Participants

Data were collected as part of two larger studies within a larger project, examining children’s math and working memory skills (Ramani et al., 2019). Participants were 403 kindergarten children (mean age = 5.4 years, 51% female) recruited from public elementary and charter schools on the east coast and west coast of the United States.

At the time of consent, parents completed a survey of demographic information. Parents reported children’s race and ethnicity, parent education level, family size, annual household income, children’s language background, and children’s level of bilingualism/trilingualism.

Thirty percent of children were African American or Black, 28% were Caucasian/White, 7% were Biracial/Mixed Race, 3% were Asian or Pacific Islander, 1% were American Indian or Alaska Native, 2% were other, and 29%, did not report race. For ethnicity, 45% of children were Hispanic/Latino, 37% were not Hispanic/Latino, 7% were other, and 11% did not report ethnicity.

Parents also reported the highest level of education for each of the child’s parents/guardians. If parents selected multiple levels of education, the highest selected level was used. For mothers, 13% had some high school coursework, 27% had a high school diploma/GED, 25% had some college coursework/vocational training, 8% had a 2-year college degree, 8% had a 4-year college degree, 10% had a postgraduate or professional degree, and 9% did not report mother’s education. For children’s other parent, 18% had some high school coursework, 38% had a high school diploma/GED, 11% had some college coursework/vocational training, 5% had a 2-year college degree, 7% had a 4-year college degree, 6% had a postgraduate or professional degree, and 15% did not report other parent’s education.

Eighty-eight percent of families reported their family size (the number of people typically residing in their household). The average reported family size was 4.42, with a range from 1 to 10.

For annual household income, 19% of families reported an annual household income less than $15,000, 23% reported an annual income of $15,000–$30,000, 13% reported an annual income of $31,000–$45,000, 8% reported an annual income of $46,000–$59,000, 6% reported an annual income of $60,000–$75,000, 5% reported an annual income of $76,000–$100,000, 5% reported an annual income of $101,000–$150,000, and 5% reported an annual income of $151,000 or more. Fifteen percent of families did not report annual household income.

Parents also reported the language children spoke the most at home. Specifically, 68% reported English, 15% reported Spanish, 3% reported English and Spanish, 1% reported Arabic, 1% reported Vietnamese, less than 1% reported Russian, less than 1% reported Turkish, less than 1% reported Albanian, less than 1% reported Japanese, and 10% did not report the language spoken at home.

In addition, parents reported their child’s level of bi/trilingualism on a scale of 1 to 5. Thirty-four percent of children were not bi/trilingual (spoke predominantly one language), 11% were weak bi/trilinguals, 10% were non-fluent bi/trilinguals, 6% were practical bi/trilinguals, and 6% were fluent bi/trilinguals. 3% of families reported mixed categories, and 30% of families did not report children’s level of bi/trilingualism.

### 2.2. Procedure

Children completed a measure of addition strategy one-on-one with an experimenter in their classroom or another room at their elementary school. Prior to participating, parents provided informed consent and children provided verbal assent.

### 2.3. Measures

#### 2.3.1. Addition strategy

The addition strategy items, procedure, and coding were adapted from commonly used measures of addition strategy (e.g., Geary et al., 2004). Children were asked to solve a series of addition problems as quickly as they could without making too many mistakes. They were told they could use whatever way was easiest for them to get an answer. In one study, problems were shown one at a time on a computer screen. In the other study, problems were shown one at a time in a printed flip book. In both studies, two sets of problems (i.e., Set A, Set B) were used and were evenly counterbalanced across participants.

Children completed one practice problem (2 + 2) with feedback and 12 test problems with no feedback. Two problems were not included in these analyses as they differed across studies from which data were collected. The remaining 11 problems were administered in both studies. These included one practice problem, six simple problems, and four complex problems (Set A: 2 + 2, 3 + 5, 8 + 4, 16 + 7, 9 + 2, 9 + 15, 6 + 4, 14 + 8, 4 + 9, 3 + 18, 5 + 2; Set B: 2 + 2, 3 + 4, 6 + 2, 9 + 3, 9 + 14, 3 + 19, 7 + 3, 16 + 8, 8 + 5, 15 + 6, 4 + 7). For the simple problems, half of the problems had sums less than or equal to ten, and half had sums greater than ten. Approximately half of each of the simple and complex problems presented the larger addend first.

For each problem, the experimenter read the problem out loud (e.g., “What is 2 plus 2?”) and recorded children’s responses as well as any observed use of problem-solving strategies. After the children responded, the experimenter asked them how they got their answers. Children’s accuracy was coded for each addition problem.

#### 2.3.2. Addition strategy coding

Strategies were coded from experimenter observations and children’s explanations of how they got their answer. Experimenters classified children’s behaviors while solving the problems as using finger or verbal counting, retrieval, decomposition, or an undetermined strategy. Finger and verbal counting strategies were further classified as Min (starting at the higher number and counting up), Max (starting at the lower number and counting up), Sum (starting at zero and counting the sum of the two numbers), or Not specified (e.g., saying numbers in a random order, random finger movements, inaudible mouth movements). If children used both finger and verbal counting, but different subcategories of counting (e.g., min finger count and max verbal count), the more sophisticated strategy was recorded (e.g., mixed min count).

If children’s descriptions of how they got their answers differed from experimenter observations (e.g., the experimenter observed finger counting and the child said they just knew it/retrieval), the experimenter’s observations were used as the strategy observed. When no strategies were observed by the experimenter, the child’s explanation was used to classify the strategy as retrieval or undetermined. Explanations including retrieval strategies (“I knew it,” “Someone told me,” “I guessed,” “I used my brain”) were classified as retrieval, and explanations including other strategies or nonsense answers (e.g., “I think it is,” “It is easy”) were classified as undetermined.

For the current study, responses were then coded based on a combination of accuracy and strategy sophistication (coding scheme adapted from Chu et al., 2018). Considering scores in this way is particularly useful because this approach takes into account problem-solving accuracy for each individual strategy used and scores values along a continuum, such that higher scores indicate correct answers solved with more sophisticated strategies, and lower scores indicate incorrect answers solved with less sophisticated strategies. The current coding scheme included 10 values, with values representing problems solved incorrectly and problems solved correctly, with increasingly sophisticated strategies (see Table 1 for values and definitions). Children’s codes were summed to get total combined strategy and accuracy scores for all problems, for simple problems, and for complex problems. The average score for each problem type was used as an outcome measure.

**Table 1:** Strategy and accuracy coding definitions.

Code	Value	Includes
Missing	0	Missing
Undetermined error	1	Error: Undetermined
Retrieval error	2	Error: Retrieval, Guessing, Count in head, Decomposition
Counting error	3	Error: Any counting strategy
Undetermined	4	Correct: Undetermined
Other count	5	Correct: Other counting
Sum/Max count	6	Correct: Sum/Max counting
Min count	7	Correct: Min counting
Advanced strategy	8	Correct: Count in head, Decomposition
Retrieval	9	Correct: Retrieval, Guessing

#### 2.3.3. Socioeconomic status

A composite consisting of household income and parent education was used as a measure of SES. First, an income-to-needs ratio was calculated by dividing the reported annual household income by the Census poverty threshold for the reported family size from the year of data collection (2016 or 2018). Because annual household income was reported on a scale of income intervals (e.g., $15,000 to $30,000), the midpoint of each family’s reported income interval (e.g., $22,500 in this example) was used as the family’s income for the calculation. Eighty-two percent of participants reported both income and family size, and family income-to-needs for those participants ranged from 0.3 to 7.9 (mean = 1.91). Family income-to-needs was positively correlated with mother’s education (*r*(304) = 0.691, *p* < 0.001) and with other parent’s education (*r*(304) = 0.695, *p* < 0.001). To create the composite of household income and parent education, the family income-to-needs ratio variable, mother’s education variable, and other parent’s education variable were each standardized. The range of values for these standardized variables was as follows: income-to-needs −0.96 to 3.56, mother’s education −1.33 to 1.97, other parent’s education −1.06 to 2.34. The total of the standardized values was used as the composite (as in prior measures; Hauser, 1994; Levine et al., 2010; Daubert et al., 2019).

#### 2.3.4. Home activities survey

Parents completed a home activities survey at the time of consent. Parents reported the frequency with which they and their child had engaged in 12 literacy and 12 math activities over the past month (adapted from LeFevre et al., 2009; Skwarchuk et al., 2014; see Table 2 for a summary of the items).

**Table 2:** Summary of home activities survey.

Item	Activity	*n*	*M*	*SD*
Item 1	Reading together	362	3.42	1.29
Item 2	Saying/singing the ABCs	347	3.13	1.46
Item 3	Counting out loud	351	3.72	1.26
Item 4	Counting by a number other than 1 (by 2’s, by 5’s, by 10’s)	354	2.47	1.65
Item 5	Noticing letters and words	357	3.89	1.18
Item 6	Counting objects	353	3.89	1.23
Item 7	Labeling letters or words	353	3.38	1.39
Item 8	Talking about how many objects are in a set (e.g., there are 5 toys in the basket)	357	3.38	1.39
Item 9	Memorizing letters/sounds or sight words	363	3.67	1.30
Item 10	Memorizing math facts	354	3.61	1.33
Item 11	Writing numbers	357	3.47	1.31
Item 12	Point to letters/words while reading	354	3.61	1.33
Item 13	Comparing numbers (e.g., “2” is bigger than “1”)	354	3.07	1.44
Item 14	Counting down (10, 9, 8, 7...)	351	2.97	1.60
Item 15	Talking about meanings of words	356	3.32	1.39
Item 16	Talking about what letters words start with	357	3.29	1.51
Item 17	Introducing new words and definitions	354	3.14	1.53
Item 18	Counting out money	347	2.30	1.48
Item 19	Asking questions when reading together	351	3.42	1.38
Item 20	Comparing amounts (e.g., 3 cookies is more than 1 cookie)	351	3.06	1.54
Item 21	Talking about letter sounds	353	3.53	1.38
Item 22	Using fingers to indicate how many	352	3.75	1.29
Item 23	Sounding out words	346	3.62	1.47
Item 24	Learning simple sums (e.g., 2 + 2)	356	3.27	1.51

Activities were rated based on the past month and rated on the following scale: (0) did not occur, (1) 1–3 times per month, (2) once per week, (3) 2–4 times per week, (4) almost daily, (5) daily, or (NA) activity is not relevant to my child.

## 3. Results

### 3.1. Preliminary analyses

Preliminary analyses were conducted in order to create meaningful composite variables from the home activities survey. An exploratory factor analysis was conducted using principal components analysis. Missing data were handled with listwise deletion, leaving a subsample of *n* = 269 participants with complete data on the home activities survey.^1^ A Velicer’s MAP test for number of components to extract indicated that 3 components should be extracted. Direct oblimin oblique rotation was used to account for overlap among components and to maximize the interpretability.

Three components were identified from this analysis, representing a basic activities factor, an advanced math activities factor, and a literacy activities factor (see Table 3 for loadings). These factors were used in subsequent analyses. As shown in Table 3, the literacy activities factor included 11 activities, such as reading together and talking about the meanings of words. The advanced math activities factor included eight activities such as learning simple sums and memorizing math facts. The basic activities factor included five items such as counting out loud and counting objects. This factor also included the saying/singing the ABC’s activity, which, while related to literacy, is also a fundamental basic skill in early development, the same way that counting is.

**Table 3:** Summary of items and factor loadings.

Item	Activity	Advanced math activities	Literacy activities	Basic activities
Item 1	Reading together		−0.838	
Item 2	Saying/singing the ABCs			0.884
Item 3	Counting out loud			0.755
Item 4	Counting by a number other than 1 (by 2’s, by 5’s, by 10’s)	0.659		
Item 5	Noticing letters and words		−0.723	
Item 6	Counting objects			0.513
Item 7	Labeling letters or words		−0.557	
Item 8	Talking about how many objects are in a set (e.g., there are 5 toys in the basket)			0.552
Item 9	Memorizing letters/sounds or sight words		−0.469	
Item 10	Memorizing math facts	0.764		
Item 11	Writing numbers	0.602		
Item 12	Point to letters/words while reading		−0.744	
Item 13	Comparing numbers (e.g., “2” is bigger than “1”)	0.631		
Item 14	Counting down (10, 9, 8, 7...)	0.482		
Item 15	Talking about meanings of words		−0.743	
Item 16	Talking about what letters words start with		−0.697	
Item 17	Introducing new words and definitions		−0.737	
Item 18	Counting out money	0.509		
Item 19	Asking questions when reading together		−0.856	
Item 20	Comparing amounts (e.g., 3 cookies is more than 1 cookie)	0.469		
Item 21	Talking about letter sounds		−0.536	
Item 22	Using fingers to indicate how many			0.426
Item 23	Sounding out words		−0.659	
Item 24	Learning simple sums (e.g., 2 + 2)	0.668		

We also conducted preliminary exploratory analyses to examine differences in average strategy use and home activities by potential covariates (gender and level of bilingualism/trilingualism). Results from t-tests indicated that there were no significant differences in strategy use (*t*(383) = 1.359, *p* = 0.175, *d* = 0.139), basic activities (*t*(359) = −1.645, *p* = 0.101, *d* = −0.173), advanced math activities (*t*(359) = 0.536, *p* = 0.592, *d* = 0.056), or literacy activities (*t*(360) = −0.581, *p* = 0.561, *d* = −0.061) as a function of children’s gender. To examine the level of bi/trilingualism, children’s level of bilingualism/trilingualism was classified into one of three groups: fluent monolingual, fluent bi/trilingual, and non-fluent bi/trilingual. Results from one-way ANOVAs indicated that there were no significant differences in strategy use (*F*(1, 268) = 0.645, *p* = 0.423), basic activities (*F*(1, 267) = 0.260, *p* = 0.611), advanced math activities (*F*(1, 267) = 1.39, *p* = 0.240), or literacy activities (*F*(1, 268) = 1.886, *p* = 0.171) based on children’s level of bilingualism. Because there were no significant differences, gender and level of bilingualism/trilingualism were not included as covariates in subsequent analyses.

### 3.2. Descriptive statistics

Table 4 shows descriptive statistics for addition strategy use, home activities, and SES.

**Table 4:** Descriptive statistics for addition strategy, home activities, and SES variables.

	*n*	Min	Max	*M*	*SD*
Average strategy use (overall)	399	0	7.7	3.21	1.48
Average strategy use (simple problems)	399	0	8.5	3.76	1.80
Average strategy use (complex problems)	399	0	7.25	2.36	1.37
Percent missing (Strategy 0)	401	0	100	3.34	12.80
Percent undetermined error (Strategy 1)	400	0	100	24.63	31.63
Percent retrieval error (Strategy 2)	400	0	100	19.68	25.79
Percent counting error (Strategy 3)	400	0	100	25.90	26.58
Percent undetermined (Strategy 4)	400	0	30	1.20	4.01
Percent other count (Strategy 5)	400	0	90	4.30	11.85
Percent sum/max count (Strategy 6)	400	0	80	8.20	11.92
Percent min count (Strategy 7)	400	0	80	5.57	11.42
Percent advanced strategy (Strategy 8)	400	0	70	3.37	10.01
Percent retrieval (Strategy 9)	400	0	50	4.05	8.32
Basic activities	363	0	5	3.58	1.05
Advanced math activities	363	0	5	2.93	1.16
Literacy activities	364	0	5	3.46	1.08
SES	303	−3.29	7.87	0.11	2.74

### 3.3. Primary analyses

#### 3.3.1. Aim 1: Structural characteristics of the home

The first aim was to examine the relations between children’s addition strategy use and SES, as a replication of previous research. Correlations between SES and children’s average strategy use for simple and complex problems and percent strategy use for the types of strategies are shown in Table 5. SES was significantly positively correlated with children’s strategy use overall and on simple and complex addition problems, such that children from higher SES backgrounds were more likely to solve addition problems accurately using more sophisticated strategies.

**Table 5:** Correlations between strategy use, home activities, and socioeconomic status.

	1.	2.	3.	4.	5.	6.	7.	8.	9.	10.	11.	12.	13.	14.	15.	16.	17.
1. Average strategy use (overall)	–																
2. Average strategy use (simple problems)	0.956**	–															
3. Average strategy use (complex problems)	0.817**	0.612**	–														
4. Percent missing (Strategy 0)	−0.167**	−0.077	−0.298**	–													
5. Percent undetermined error (Strategy 1)	−0.674**	−0.649**	−0.541**	−0.151**	-												
6. Percent retrieval error (Strategy 2)	−0.240**	−0.235**	−0.185**	−0.109*	−0.197**	–											
7. Percent counting error (Strategy 3)	0.199**	0.163**	0.217**	−0.097	−0.443**	−0.385**	–										
8. Percent undetermined (Strategy 4)	0.101*	0.107*	0.061	0.001	−0.002	−0.083	−0.144**	–									
9. Percent other count (Strategy 5)	0.340**	0.266**	0.394**	−0.045	−0.218**	−0.173**	−0.045	0.086	–								
10. Percent sum/max count (Strategy 6)	0.419**	0.434**	0.277**	−0.024	−0.373**	−0.221**	0.252**	−0.039	−0.071	–							
11. Percent min count (Strategy 7)	0.521**	0.507**	0.410**	−0.045	−0.260**	−0.186**	−0.01	0.018	0.004	0.114*	–						
12. Percent advanced strategy (Strategy 8)	0.516**	0.473**	0.461**	−0.031	−0.201**	−0.083	−0.153**	0.024	0.112*	−0.01	0.067	–					
13. Percent retrieval (Strategy 9)	0.415**	0.458**	0.220**	−0.006	−0.117*	−0.008	−0.208**	0.124*	0.069	−0.126*	0.028	0.103*	–				
14. Basic activities	0.033	0.019	0.051	−0.066	−0.055	0.011	0.077	0.014	0.023	−0.058	0.095	−0.049	−0.006	–			
15. Advanced math activities	0.182**	0.172**	0.154**	−0.06	−0.162**	−0.041	0.109*	0.06	0.075	0.028	0.139**	0.02	0.077	0.760**	–		
16. Literacy activities	0.235**	0.210**	0.222**	−0.139**	−0.169**	−0.044	0.099	0.039	0.071	0.111*	0.197**	0.037	0.045	0.715**	0.771**	–	
17. SES	0.361**	0.308**	0.369**	−0.068	−0.254**	−0.051	−0.007	0.071	0.150**	0.235**	0.227**	0.165**	0.019	−0.065	−0.045	0.304**	–

*p < 0.05, **p < 0.01.

We also conducted regression analyses predicting average strategy use from SES. Results indicated that SES was a significant predictor of children’s strategy sophistication overall, for simple addition problems, and for complex addition problems (Table 6). Overall, we found that SES accounted for 13% of the variance in children’s average strategy use on the addition problems.

**Table 6:** Summary of regressions predicting average strategy use from SES.

	Average strategy use (overall)			Average strategy use (simple problems)			Average strategy use (complex problems)		
Variable	*β*	*t*	*p*	*β*	*t*	*p*	*β*	*t*	*p*
SES	0.204	6.681	<0.001***	0.209	5.572	<0.001***	0.196	6.847	<0.001***
	*R*^2^ = 0.131			*R*^2^ = 0.095			*R*^2^ = 0.136		
	*F*(1, 297) = 44.630, *p* = <0.001***			*F*(1, 297) = 31.051, *p* = <0.001***			*F*(1, 297) = 46.876, *p* = <0.001***		

***p < 0.001.

As part of Aim 1, we also examined more in-depth differences between lower- and higher-income groups in strategy sophistication. In these analyses, we used income as a measure of SES, to be able to compare with previous research (e.g., Laski et al., 2016). Specifically, to examine lower- and higher-income groups, we used an income-to-needs ratio of 1 as a threshold, comparing less than 1 with greater than or equal to 1 (e.g., Duncan et al., 1994; Dearing et al., 2001).

T-tests were used to compare strategy use for lower (n = 140) and higher (n = 190) income groups. For average strategy use, results indicated that there were significant differences in strategy use overall (*M*_low_ = 2.87, *M*_high_ = 3.53; *t*(324) = −3.92, *p* < 0.001, *d* = −0.439), on simple problems (*M*_low_ = 3.37, *M*_high_ = 4.12; *t*(324) = −3.75, *p* < 0.001, *d* = −0.420), and on complex problems (*M*_low_ = 2.13, *M*_high_ = 2.63; *t*(324) = −3.19, *p* = 0.002, *d* = −0.357), such that children from higher-income backgrounds were more accurate and used more sophisticated strategies than children from lower-income backgrounds.

**Figure 1:**
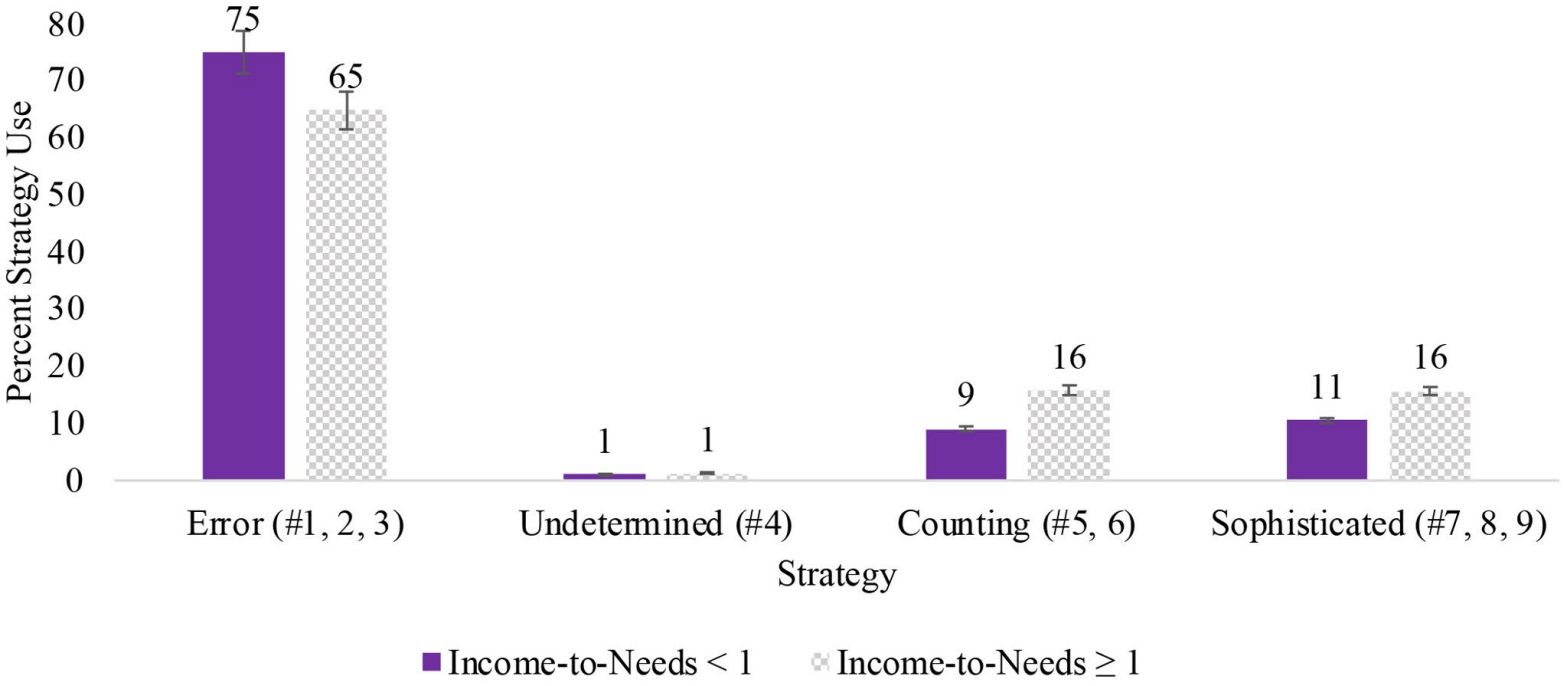
Strategy use by lower- and higher-income groups.

We also compared differences in strategy use for use of the coded strategies (Figure 1). For interpretability, strategies are grouped as Error (strategies 1, 2, and 3), Undetermined (strategy 4), Counting (strategies 5 and 6), and Sophisticated (strategies 7, 8, and 9). We found the same pattern of results as average strategy use. Specifically, results indicated that there were significant differences in Error (*M*_low_ = 75.18, *M*_high_ = 64.95; *t*(325) = 3.25, *p* = 0.001, *d* = 0.364), Counting (*M*_low_ = 9.06, *M*_high_ = 15.90; *t*(325) = −3.69, *p* < 0.001, *d* = −0.413), and Sophisticated (*M*_low_ = 10.58, *M*_high_ = 15.69; *t*(325) = −2.44, *p* = 0.015, *d* = −0.273) strategy use, such that children from higher-income backgrounds were more likely to use counting and sophisticated strategies and less likely to have errors. There were no significant differences in Undetermined (*M*_low_ = 1.15, *M*_high_ = 1.28; *t*(325) = −0.273, *p* = 0.785, *d* = −0.030) strategy use, which was infrequently used overall.

#### 3.3.2. Aim 2: Home learning environment

The second aim was to examine how children’s engagement in activities at home related to their addition strategy sophistication. Correlations and regressions were used to examine these relations. Table 5 shows correlations between home activities composites and average strategy use for simple and complex problems and percent strategy use for the types of strategies. Overall, basic activities were not significantly correlated with average strategy use, however, advanced math and literacy activities were significantly correlated with average strategy use for both simple and complex arithmetic problems. In examining relations with specific strategy types, we found that basic activities were not significantly related to any individual strategy types. Advanced math activities were significantly negatively related to Undetermined Error and significantly positively related to Counting Error and Min Count. Literacy activities were significantly negatively related to Undetermined Error and significantly positively related to Sum/Max Count and Min Count.

Results from regression analyses predicting average strategy use from home activities composites (Table 7) indicate that basic activities significantly negatively predicted strategy use and advanced math and literacy activities significantly positively predicted strategy use overall and for simple addition problems. For the complex addition problems, basic activities negatively predicted strategy use and literacy activities positively predicted strategy use, but advanced math activities did not.

**Table 7:** Summary of regressions predicting average strategy use from home activities.

	Average strategy use (overall)			Average strategy use (simple problems)			Average strategy use (complex problems)		
Variable	*β (SE)*	*t*	*p*	*β (SE)*	*t*	*p*	*β (SE)*	*t*	*p*
Basic activities	−0.514 (0.117)	−4.387	<0.001***	−0.625 (0.142)	−4.403	<0.001***	−0.348 (0.111)	−3.124	0.002**
Advanced math activities	0.238 (0.116)	2.049	0.041*	0.319 (0.141)	2.276	0.023*	0.114 (0.110)	1.038	0.299
Literacy activities	0.490 (0.116)	4.224	<0.001***	0.527 (0.141)	3.753	<0.001***	0.434 (0.110)	3.938	<0.001***
	*R*^2^ = 0.104			*R*^2^ = 0.095			*R*^2^ = 0.074		
	*F*(3, 356) = 13.79, *p* = <0.001***			*F*(3, 356) = 12.41, *p* = <0.001***			*F*(3, 356) = 9.528, *p* = <0.001***		

*p < 0.05, **p < 0.01, ***p < 0.001.

As part of Aim 2, we also examined more in-depth differences between lower- and higher-income groups in reported engagement in home activities. As in Aim 1, we used income as a measure of SES, and used an income-to-needs ratio of 1 as a threshold, comparing less than 1 with greater than or equal to 1.

T-tests were used to compare home activities for lower- and higher-income groups. For home activities, results indicated that there were significant differences in literacy activities (*M*_low_ = 3.09, *M*_high_ = 3.62; *t*(320) = −4.52, *p* < 0.001, *d* = −0.510), such that children from higher-income backgrounds engaged in literacy activities at home more frequently than children from lower-income backgrounds. There were no significant differences in basic activities (*M*_low_ = 3.53, *M*_high_ = 3.50; *t*(319) = 0.262, *p* = 0.794, *d* = 0.030) or advanced math activities (*M*_low_ = 2.86, *M*_high_ = 2.86; *t*(319) = 0.014, *p* = 0.989, *d* = 0.002).

#### 3.3.3. Aim 3: Structural characteristics × home learning environment

The third aim was to examine if children’s engagement in home activities moderated the relations between SES and children’s addition strategy use. Separate analyses were conducted for each activity type: basic, advanced math, and literacy activities. Table 8 shows results from regression models predicting average strategy use. Results indicated that advanced math and literacy activities significantly predicted strategy use overall as well as for the simple and complex problems. However, none of the SES x activities interactions were significant for any activity type indicating that home activities did not serve as a moderator between SES and children’s addition strategy use.

**Table 8:** Summary of regression models predicting average strategy use.

	Average strategy use (overall)			Average strategy use (simple problems)			Average strategy use (complex problems)		
	*β*	*t*	*p*	*β*	*t*	*p*	*β*	*t*	*p*
***Basic activities***									
SES	0.150	1.563	0.119	0.173	1.469	0.143	0.116	1.280	0.202
Activities	0.081	1.013	0.312	0.065	0.657	0.512	0.106	1.410	0.160
SES × Activities	0.016	0.572	0.567	0.009	0.274	0.785	0.026	0.988	0.324
	*R*^2^ = 0.129			*R*^2^ = 0.090			*R*^2^ = 0.145		
	*F*(3,288) = 14.32, *p* < 0.001**			*F*(3,288) = 9.547, *p* < 0.001**			*F*(3,288) = 16.28, *p* < 0.001**		
***Advanced math activities***									
SES	0.155	1.954	0.052	0.195	1.997	0.0468*	0.095	1.272	0.205
Activities	0.256	3.490	<0.001**	0.283	3.146	0.002**	0.214	3.093	0.002**
SES × Activities	0.019	0.674	0.501	0.004	0.131	0.896	0.040	1.529	0.127
	*R*^2^ = 0.162			*R*^2^ = 0.119			*R*^2^ = 0.169		
	*F*(3,288) = 18.50, *p* < 0.001**			*F*(3,288) = 12.98, *p* < 0.001**			*F*(3,288) = 19.45, *p* < 0.001**		
***Literacy activities***									
SES	0.177	1.318	0.189	0.233	1.412	0.159	0.093	0.734	0.464
Activities	0.204	2.356	0.019*	0.219	2.067	0.0396*	0.180	2.207	0.028*
SES × Activities	−0.001	−0.014	0.989	−0.016	−0.367	0.714	0.022	0.679	0.498
	*R*^2^ = 0.144			*R*^2^ = 0.106			*R*^2^ = 0.149		
	*F*(3,289) = 16.16 *p* < 0.001**			*F*(3,289) = 11.36, *p* < 0.001**			*F*(3,289) = 16.94, *p* < 0.001**		

*p < 0.05, **p < 0.001.

## 4. Discussion

The goal of the current study was to examine the role of home environmental factors in children’s accuracy and strategy sophistication while solving numerical addition problems. We considered both structural characteristics of the home (e.g., SES) and the home learning environment (e.g., engagement in math and literacy activities at home). Findings indicated that SES related to children’s strategy sophistication (Aim 1), and that frequency of engaging in advanced math and literacy activities at home predicted strategy sophistication (Aim 2); however, in contrast to our expectations, engaging in activities at home did not moderate the relations between SES and strategy sophistication (Aim 3).

### 4.1. SES and strategy use

Previous research has found that problem-solving accuracy and strategy use vary for children from different socioeconomic backgrounds. Specifically, studies have shown that children from lower-income backgrounds have lower accuracy and use less sophisticated strategies when solving problems than children from higher-income backgrounds (Laski et al., 2016). We replicated these results in the current study, finding that children from higher SES backgrounds (based on income and parent education) were more likely to solve addition problems accurately using more sophisticated strategies. This pattern was consistent for both simple addition problems and complex addition problems. Overall, we found that SES explained 13% of the variance in strategy sophistication. For comparisons based on only income, we found that children from higher-income backgrounds were more likely to use counting and sophisticated strategies and less likely to have errors than children from lower-income backgrounds. Specifically, children from lower-income backgrounds had errors on 75% of problems, compared to 65% of problems for children from higher-income backgrounds. Many factors, including differences in access to resources and learning opportunities, may contribute to these differences. In order to get a better understanding of the factors impacting these differences, we further examined the role of the home learning environment as one of the variables driving the association between strategy use and SES. Understanding the role of these home factors is important for developing interventions and making recommendations for ways to support children in their development of math and problem-solving skills.

### 4.2. Home learning environment and strategy use

We examined the home learning environment in relation to children’s addition accuracy and strategy sophistication, as previous research has shown that engagement in math and literacy activities at home positively relates to children’s math skills. Overall, our results showed variability in families’ engagement in each type of activity at home. In examining differences between lower- and higher-income groups, we found that children from higher-income backgrounds engaged in more literacy activities than children from lower-income backgrounds, but that there were no differences between groups in basic activities or advanced math activities. Previous studies of the home math environment show inconsistent patterns, with some studies finding that children from higher-income backgrounds engage in more math activities at home than children from lower-income backgrounds (DeFlorio and Beliakoff, 2015) and that SES relates to engagement in math activities at home (Susperreguy et al., 2020), some finding that there are no significant relations between home math activities and SES (Hart et al., 2016; De Keyser et al., 2020), and other studies finding that home math activities relate to parent education-based measures of SES but not income-based measures of SES (Muñez et al., 2021). In the current study, it is possible that while there were no differences between groups in the frequency of engaging in basic activities and advanced math activities, there could be potential differences in other aspects of engagement in the activities, such as the type and quality of parent–child interactions during the activities.

In examining relations between home learning activities and addition strategy sophistication, we found different patterns of results for each activity type. Engaging in literacy activities was correlated with strategy use and significantly predicted average strategy use for simple and complex problems. This finding is consistent with prior work that shows positive relations between literacy activities and math performance (Anders et al., 2012; Manolitsis et al., 2013), and extends previous findings by examining these activities specifically in relation to arithmetic and problem-solving strategy sophistication. Previous research has suggested that engaging in literacy activities supports math development through vocabulary and language skills and through relations of the home literacy and home math environments (Anders et al., 2012; Manolitsis et al., 2013; Napoli and Purpura, 2018). In the current study, we also found that the frequency of engaging in literacy activities and advanced math activities at home was related. In addition, as previous research has indicated that language and phonological skills relate to arithmetic performance (Vukovic and Lesaux, 2013; Liu et al., 2020), it is also possible that engaging in literacy activities at home that support development of phonological skills can support children’s development of arithmetic strategy use through these skills as well.

Engaging in basic activities did not correlate with strategy use and negatively predicted average strategy use for simple and complex addition problems. In contrast, engaging in advanced math activities was correlated with strategy use and positively predicted average strategy use. This is consistent with other research showing that advanced but not basic math activities are predictive of kindergarten children’s performance on a standardized math test (Muñez et al., 2021). One reason for this difference may be the types of skills that are practiced during each type of activity. In the current study, advanced math activities included activities that were more directly related to arithmetic and problem-solving (e.g., learning simple sums, memorizing math facts, and comparing numbers) than basic activities, which were more focused on counting and cardinality skills (e.g., counting out loud, talking about how many objects are in a set). Previous research has shown that there can be specificity in the relations between home activities and math skills. For example, Leyva et al. (2021) found that the frequency of engaging in adding/subtracting activities at home predicted 4-year-old children’s performance on addition and subtraction story problems. The current study adds to these findings by examining not only accuracy in problem solving, but strategy use during problem solving as well. In summary, our findings considering the different categories of home activities in relation to strategy use suggest that engaging in advanced math activities and literacy activities may support children’s arithmetic and problem-solving skill development more than engaging in basic activities. These findings have implications for family engagement. Specifically, although basic activities (e.g., counting activities) are also important for children’s early number skills, it is possible that engaging in activities around more advanced math skills (e.g., comparing numbers and quantities, applying basic number skills) may be particularly important for supporting more advanced math skills, such as arithmetic and use of sophisticated problem-solving strategies.

### 4.3. SES, home learning environment, and strategy use

We examined home activities as a potential moderator of the relations between SES and strategy use. As expected, our results indicated that engaging in advanced math activities and literacy activities at home predicted strategy use above and beyond SES. These results provide further evidence that home activities are important to consider in relation to children’s addition accuracy and strategy sophistication, and that certain types of activities may relate to children’s arithmetic skills more than others.

Contrary to our predictions, however, relations between SES and strategy use did not vary based on the frequency of engaging in activities at home. Previous studies have found varying relations between SES, the home learning environment, and children’s math skills. For example, Dearing et al. (2012) found that general home learning investments (e.g., encouraging children to develop hobbies, the child having a desk or special place for reading or studying) mediated the relations between SES and math activities, and that math activities mediated the relations between general home learning investments and arithmetic performance. Another study examining relations of the home learning environment and math achievement found that SES moderated the relations between math achievement and general learning activities (e.g., play games or do puzzles, talk about nature or do science projects, play sports and build things together) and math achievement and between reading learning activities (e.g., frequency of looking at picture books) and math achievement, with results indicating that the relations between activities and achievement were stronger for children from higher SES backgrounds (Galindo and Sonnenschein, 2015). In addition, results from a meta-analysis found that overall, SES did not moderate relations of the home math environment and children’s math achievement, however, there were differences in the effects based on the SES of the samples, with results indicating that the relation between direct activities and math was stronger for children from lower SES backgrounds than children from higher SES backgrounds (Daucourt et al., 2021).

Results from the current study add to these previous findings by indicating that engaging in advanced math activities and literacy activities at home predicted strategy use above and beyond SES. In the current study, we did not test if home activities mediated the relations of SES and strategy use, because SES did not predict engagement in basic or advanced math activities at home. Further, as described above, there were no differences between lower- and higher-income groups in basic activities or advanced math activities in the current sample. It is possible that other differences could contribute to the pattern of results. For example, the current measure of home learning activities focused on frequency of engaging in activities at home. It is possible that differences in how parents and children engage in activities together (e.g., the types of talk parents and children engage in, parent–child social engagement and interactions during the activities; and attitudes toward and enjoyment of the activities; Vandermaas-Peeler et al., 2009; Vandermaas-Peeler and Pittard, 2014) may impact relations of SES and strategy use differently than the frequency of engaging in activities together.

### 4.4. Limitations and future directions

The current study has several limitations and directions for future research. First, it is important to note the various ways SES is measured in the literature. Previous studies have used measures of SES including only income (Hart et al., 2016; Laski et al., 2016), income-to-needs calculated with income and family size (Dearing et al., 2012); school-based income-related variables (DeFlorio and Beliakoff, 2015; De Keyser et al., 2020), only parent education (Susperreguy et al., 2020) and a combination of income, parent education, and parent occupation (Galindo and Sonnenschein, 2015). Consistent with prior studies (e.g., Hauser, 1994; Levine et al., 2010; Daubert et al., 2019), the current study used a composite of income (income-to-needs calculated with annual household income and reported family size) and parent education (highest levels of education attained by the child’s mother and other parent). It is possible that examining different aspects of socioeconomic status could influence results, as it is possible that different components of SES may relate to home learning activities and children’s arithmetic skills differently.

It is also important to consider measurement of the home learning environment. The current study used parent-reported frequency of engagement in math and literacy activities at home. While this is a common method for measuring the home environment (Mutaf-Yıldız et al., 2020; Hornburg et al., 2021), other methods, such as observing parent–child engagement in activities at home, could provide additional information about the relations between home activities and addition strategy sophistication. In addition, the current measure primarily focused on formal/direct activities (Skwarchuk et al., 2014), rather than informal/indirect activities (such as playing math board or card games, singing counting songs, making up rhymes in songs; Skwarchuk et al., 2014.). As informal activities are also important for early math development and relate to children’s math performance (Niklas and Schneider, 2014; Mutaf Yıldız et al., 2018; Zhang et al., 2020), future studies could examine these types of activities in relation to children’s addition strategy use as well.

Further, the current study did not include measures to examine relations with other aspects of the home learning environment, such as parent attitudes and beliefs about math and literacy. As previous research indicates that parent attitudes and beliefs about math (e.g., importance of math, math anxiety, expectations for children’s math learning) relate to children’s math performance (Elliott and Bachman, 2018), it is possible that these factors could influence children’s arithmetic and strategy sophistication as well.

Finally, in the current study, the number of problems that children answered correctly versus incorrectly was not evenly distributed. Children answered the majority of the arithmetic problems incorrectly. Future studies could examine relations between SES, home activities, and strategy sophistication in a sample with a more even distribution of correct and incorrect responses to see if results are consistent when children have higher accuracy in problem solving. In addition, future work could further examine the types of errors children made, to understand children’s problem solving more in-depth. For example, studies could examine the absolute error as well as if errors fall into patterns which could indicate usage of other strategy types (e.g., an addend plus one, naming an addend; Laski et al., 2016). Examining these would allow for more understanding of relations between strategy use and error type as well as relations between SES, home activities, and addition strategy use.

### 4.5. Conclusion

The current study examined relations between SES, home math and literacy activities, and addition strategies. The study addressed a gap in the literature by examining these aspects of children’s home environments in relation to both accuracy and strategy sophistication during an arithmetic problem-solving task. Findings indicated that SES related to strategy sophistication, and that engaging in basic activities negatively predicted strategy sophistication and engaging in advanced math and literacy activities positively predicted strategy sophistication. These results suggest that family engagement in activities at home may promote early arithmetic skills, and that the role of home environmental characteristics should be considered in children’s arithmetic strategy use and performance over development. As children’s early strategy use relates to later math and problem-solving abilities, understanding factors that influence strategy use is important for children’s math development and achievement.

## Data availability statement

The raw data supporting the conclusions of this article will be made available by the authors, without undue reservation.

## Ethics statement

The studies involving human participants were reviewed and approved by the University of Maryland, College Park Institutional Review Board, and University of California, Irvine Institutional Review Board. Written informed consent to participate in this study was provided by the participants’ legal guardian/next of kin.

## Author contributions

MD: conceptualization, formal analysis, writing – original draft. SJ: conceptualization, writing – review and editing, funding acquisition; GR: conceptualization, writing – original draft, supervision, funding acquisition. All authors contributed to and approved the manuscript.

## Funding

This research was supported by the National Science Foundation Awards DRL 1561447 and DRL 1561404 to GR and SJ.

## Conflict of interest

SJ has an indirect financial interest in the MIND Research Institute, Irvine, CA, whose interests are related to this work.

The remaining authors declare that the research was conducted in the absence of any commercial or financial relationships that could be construed as a potential conflict of interest

## Publisher’s note

All claims expressed in this article are solely those of the authors and do not necessarily represent those of their affiliated organizations, or those of the publisher, the editors and the reviewers. Any product that may be evaluated in this article, or claim that may be made by its manufacturer, is not guaranteed or endorsed by the publisher.

## Footnotes

^1^The subsample of participants with complete data on the home activities survey was used for these preliminary analyses. The full sample of participants was used for all primary analyses.
